# Relationship and Interconversion Between Superhydrophilicity, Underwater Superoleophilicity, Underwater Superaerophilicity, Superhydrophobicity, Underwater Superoleophobicity, and Underwater Superaerophobicity: A Mini-Review

**DOI:** 10.3389/fchem.2020.00828

**Published:** 2020-09-10

**Authors:** Jiale Yong, Qing Yang, Xun Hou, Feng Chen

**Affiliations:** ^1^State Key Laboratory for Manufacturing System Engineering and Shaanxi Key Laboratory of Photonics Technology for Information, School of Electronic Science and Engineering, Xi'an Jiaotong University, Xi'an, China; ^2^School of Mechanical Engineering, Xi'an Jiaotong University, Xi'an, China

**Keywords:** superhydrophilicity, superhydrophobicity, underwater superoleophilicity, underwater superoleophobicity, underwater superaerophilicity, underwater superaerophobicity

## Abstract

Superwetting surfaces have received increasing attention because of their rich practical applications. Although various superwettabilities are independently achieved, the relationship between those superwettabilities is still not well-clarified. In this mini-review, we show that superhydrophilicity, underwater superoleophilicity, underwater superaerophilicity, superhydrophobicity, underwater superoleophobicity, and underwater superaerophobicity can be obtained on a same structured surface by the combination of hierarchical surface microstructures and proper chemistry. The relationship and interconversion between the above-mentioned different superwettabilities are also well-discussed. We believe that the current discussion and clarification of the relationship and interconversion between different superwettabilities has important significance in the design, fabrication, and applications of various superwetting materials.

## Introduction

As three common states of matter, solid, liquid, and gas form different kinds of solid/liquid/gas interfaces. The materials with extreme wettability have received increasing attention because of their wide practical applications in waterproof coating (Yong et al., 2017c), anti-icing/snowing/fogging (Lv et al., 2014; Kreder et al., 2016; Chu et al., 2019), self-cleaning coating (Nishimoto and Bhushan, 2013; Yong et al., 2013c, 2014b; Ragesh et al., 2014), the manipulation of small droplets (Wang et al., 2011; Yong et al., 2013b, 2015b), corrosion resistance (Pan et al., 2013; Zhan et al., 2018), oil/water separation (Xue et al., 2014; Wang B. et al., 2015; Yong et al., 2016a,b, 2018e, 2019c; Bian et al., 2020), fog collection (Zhang et al., 2017), cell engineering (Stratakis et al., 2011; Shen et al., 2012), anti-biological adhesion (Genzer and Efimenko, 2006; Yong et al., 2018f), drag reduction in water (Shi et al., 2007), lab on a chip (Kwon et al., 2007; Vitale et al., 2013), microfluidic system (Songok et al., 2014; Wang S. et al., 2015), liquid patterning (Jokinen et al., 2008; Yong et al., 2015c), enhanced buoyancy (Yong et al., 2014c; Zhan et al., 2019), submarine gas collection (Yong et al., 2018c,d). After billions of years of evolution, creatures in nature have nearly perfect structure and function. Wherein, many organisms have evolved special surface wettability. For example, lotus leaf has the self-cleaning function (Barthlott and Neinhuis, 1997), water strider can walk on water surface (Gao and Jiang, 2004), the butterfly can shake off raindrops and fly in the rain (Zheng et al., 2007), the eyes of mosquito can repel fog (Gao et al., 2007), the fish scale cannot be polluted by oil in water (Liu et al., 2009; Yong et al., 2018b), and desert beetle, cacti, and spider silk have the capacity of harvesting water in dry air (Parker and Lawrence, 2001; Zheng et al., 2010; Ju et al., 2012). It is demonstrated that the surface wettability is primarily determined by the surface composition and the surface morphology of a solid substrate (Yong et al., 2013a; Bellanger et al., 2014; Jiang et al., 2015; Wen et al., 2015; Su et al., 2016; Bai et al., 2020). The study related to the surface wettability becomes a current research focus. Inspired by animals and plants in nature, various kinds of superwettabilities have been achieved by different microfabrication methods, such as superhydrophobicity, superhydrophilicity, underwater superoleophobicity and superoleophilicity, and underwater superaerophobicity and superaerophilicity (Teisala et al., 2014; Tian et al., 2014; Yong et al., 2014a, 2019b; Wang J. N. et al., 2015; Liu et al., 2017). Water droplet, oil droplet, or gas bubble on the material surfaces with superhydrophilicity, superoleophilicity, or superaerophilicity has a contact angle (CA) <10°, while it has a CA larger than 150° on the material surfaces with superhydrophobicity, superoleophobicity, or superaerophobicity, respectively (Tian et al., 2014; Wen et al., 2015; Yong et al., 2015a, 2017a,c; Su et al., 2016; Liu et al., 2017). Although these superwettabilities are independently achieved, the relationship between different superwettabilities is still not well-discussed. The clear relationship between different superwettabilities is important for the design of various superwetting materials and the interconversion between different superwettabilities.

In this review, the relationship and the interconversion between different superwettabilities are discussed and summarized. Taking the hydrophilic Al substrate and the hydrophobic polydimethylsiloxane (PDMS) substrate as the examples, we show that various kinds of superwettabilities can be obtained on the same structured surface. The formation mechanism of different superwettabilities and their interconversion are well-discussed and clarified.

## Achievement of Various Superwettabilities

**Figure 1:**
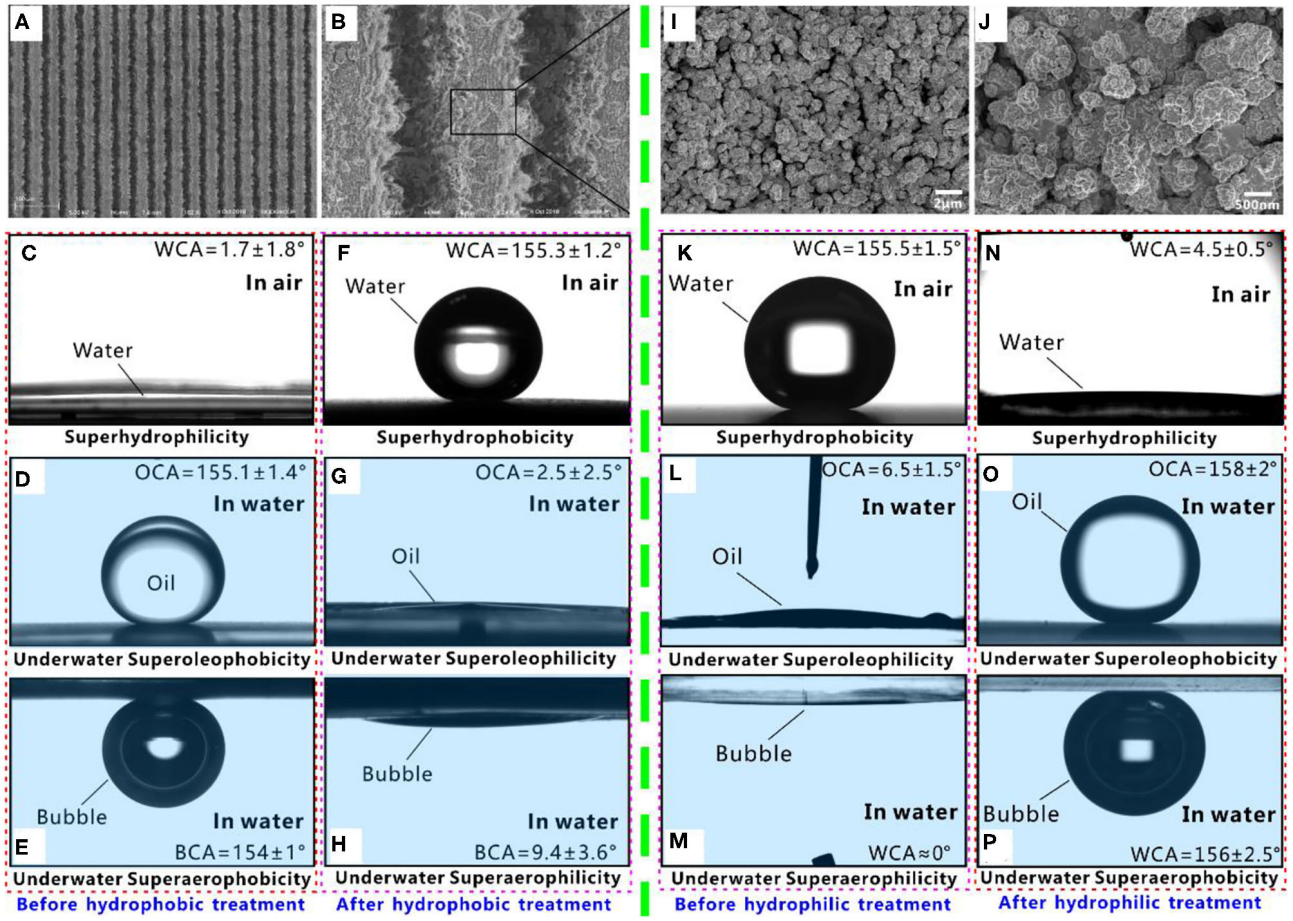
Different superwettabilities on the structured surface. **(A,B)** SEM images of the hydrophilic Al substrate with a hierarchical surface microstructure. **(C,F)** Water droplet on the structured Al surface in air. **(D,G)** Underwater oil droplet on the structured Al surface. **(E,H)** Underwater gas bubble on the structured Al surface. The samples in **(C–E)** are originally structured Al surface, while the samples in **(F–H)** are further treated by hydrophobic modification. **(I,J)** SEM images of the hydrophobic PDMS substrate with a hierarchical surface microstructure. **(K,N)** Water droplet on the structured PDMS surface in air. **(L,O)** Underwater oil droplet on the structured PDMS surface. **(M,P)** Underwater gas bubble on the structured Al surface. The samples in **(K–M)** are originally structured PDMS surface, while the samples in **(N–P)** are further treated by hydrophilic modification. Reproduced from Yong et al. (2019a) with the permission of Guo et al. Reproduced from Yong et al. (2017b) with the permission of Chen et al.

Superwettability can be designed by combining proper surface microstructures and chemistry (Yong et al., 2013a, 2017c; Bellanger et al., 2014; Jiang et al., 2015; Wen et al., 2015; Su et al., 2016; Bai et al., 2020). Al is a typical hydrophilic substrate. Figures 1A,B shows the scanning electron microscopy (SEM) images of the Al surface with rough surface microstructure (Yong et al., 2019a). The surface microstructure is created by laser ablation. There are periodic microgrooves with a width of ~35 μm, a depth of ~21 μm, and a period of 40 μm forming on the Al surface. The top of the ridges between the microgrooves is randomly coated with rich nanoparticles. A small water droplet spreads out on the structured Al surface after touching the surface, with a final water CA (WCA) of 1.7° (Figure 1C). The surface microstructure is fully wet by water, so the rough Al surface shows superhydrophilicity. The underwater wettability of such a superhydrophilic Al surface is investigated by immersing the sample in water. Oil droplets can maintain a ball-like shape on the sample and the oil CA (OCA) is measured to be 155.1° in a water medium (Figure 1D). Once the sample is tilted by 1.9°, the oil droplet can roll away freely, so the sliding angle (SA) is only 1.9°. The result reveals that the rough Al surface exhibits underwater superoleophobicity and very low adhesion to oil. Such an underwater superoleophobic surface has an excellent oil-repellent ability in a water medium. The behavior of the bubble on the superhydrophilic Al surface in water is similar to that of underwater oil droplets. Underwater superaerophobicity is exhibited by the structured Al surface. The bubble on the sample surface has a bubble CA (BCA) of 154° (Figure 1E) and SA of 0.5° in water. Therefore, a hierarchical rough Al substrate simultaneously has superhydrophilicity, underwater superoleophobicity, and underwater superaerophobicity.

Fluoroalkylsilane modification is usually adopted to lower the surface energy of a material. The fluoroalkylsilane treatment switches the structured Al surface from a superhydrophilic state to a superhydrophobic state. The water droplet on the resultant surface has a WCA of 155.3° (Figure 1F) and can roll off easily with a SA of 6.3°. The fluoroalkylsilane-modified rough Al surface exhibits ultralow adhesive superhydrophobicity and excellent repellence to water. As the superhydrophobic surface is dipped into water, a mirror-like reflectance appears on the sample surface, because a layer of air persists the superhydrophobic surface and the water (Larmour et al., 2007; Zhao et al., 2010). When an oil droplet touches the microstructure of the superhydrophobic surface in water, it will spread out along the sample surface and wet the surface microstructure. The OCA is as low as 2.5° to this oil droplet, indicating that the surface shows superoleophilicity underwater (Figure 1G). Similar to the underwater oil wettability, if a small bubble is dispensed on the superhydrophobic Al surface in water, it will also spread out and like being absorbed by the surface, with the BCA of 9.4° (Figure 1H). The surface exhibits underwater superaerophilicity to bubbles. Therefore, the fluoroalkylsilane-modified structured Al surface simultaneously has superhydrophobicity, underwater superoleophilicity, and underwater superaerophilicity.

Different from the inherently hydrophilic Al substrate, the PDMS is a kind of intrinsic hydrophobic substrate. Figures 1I,J shows the SEM images of a structured PDMS surface (Yong et al., 2017b). The surface texture is also induced by laser microfabrication. The structured surface is coated with a large number of microscale coral-like structures with several micrometers in size. The surface of the microcorals is further decorated with rich nanoscale protrusions. The hierarchical rough microstructure endows the PDMS surface with excellent superhydrophobicity. The water droplet on the structured surface has a WCA of 155.5° (Figure 1K) and a SA of 2°. In a water medium, when an oil droplet or a bubble is released onto the superhydrophobic PDMS substrate, the oil droplet or a bubble can spread out immediately and be completely absorbed by the sample surface. The measured OCA and the BCA are only 6.5° (Figure 1L) and ~0° (Figure 1M), respectively. Therefore, the underwater superoleophilicity and superaerophilicity are also exhibited by the structured PDMS surface. The surface energy of the PDMS can be increased by short-time oxygen plasma irradiation (Wu et al., 2011; Cai et al., 2014). Oxygen plasma irradiation switches the structured PDMS surface from a superhydrophobic state to a superhydrophilic state. The water droplet can fully wet the surface with a WCA of 4.5° (Figure 1N). Such a superhydrophilic PDMS surface has great repellence to both oil droplets and gas bubbles in water. Underwater oil droplet and bubble have a spherical shape on such PDMS surface, with the OCA of 158° (Figure 1O) and BCA of 156° (Figure 1P), respectively. Both oil droplets and bubbles can easily roll away from a 3° tilted sample surface (SA = 3°). Therefore, the superhydrophilic PDMS surface also exhibits underwater superoleophobicity and superaerophobicity.

## Relationship Between Different Superwettabilities

**Figure 2:**
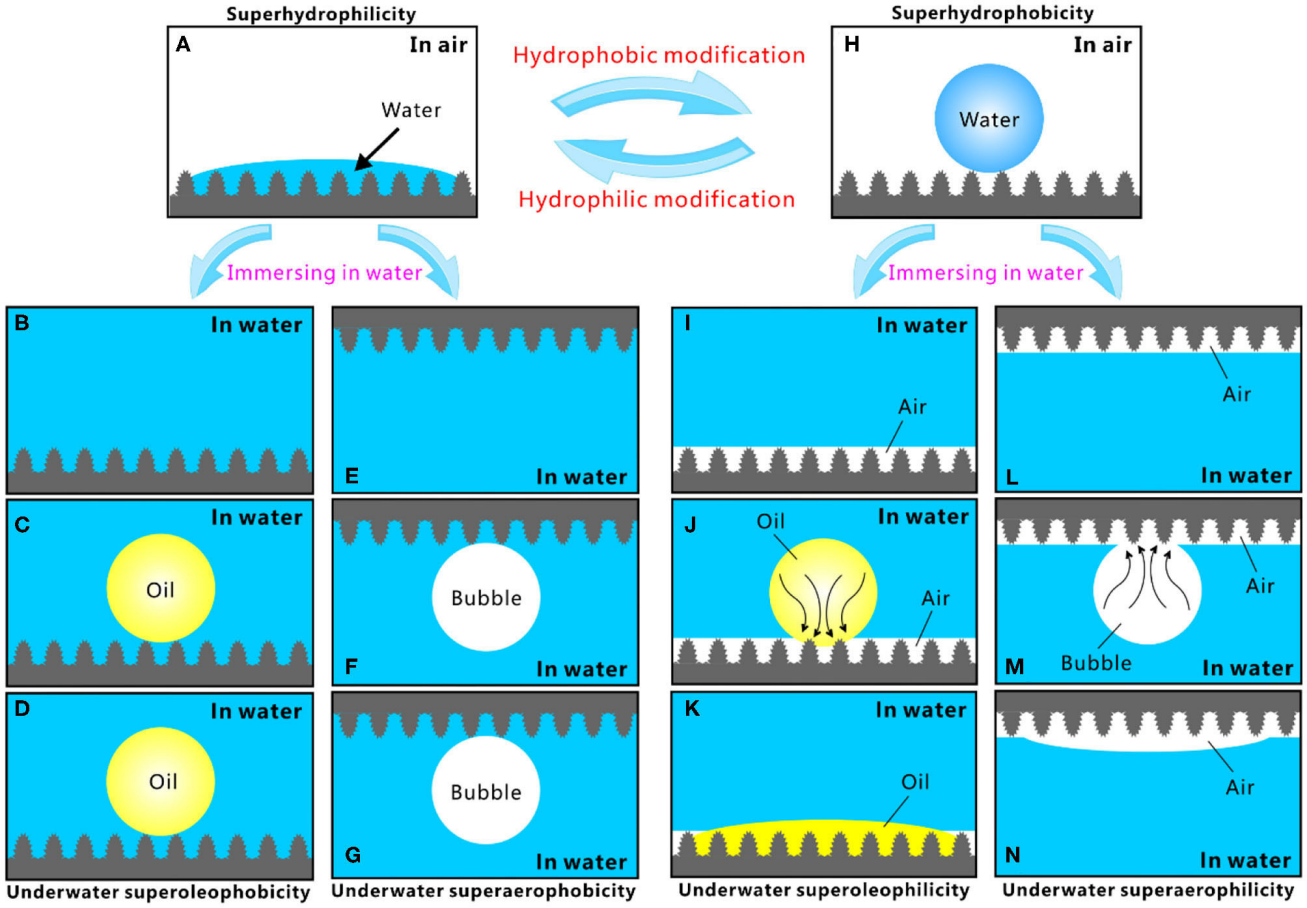
Relationship and interconversion of different superwettabilities. **(A)** Water droplet on the superhydrophilic microstructure. **(B,E)** Immersion of the superhydrophilic surface in water. **(C)** Placing an oil droplet on the superhydrophilic microstructure underwater. **(D)** Variation of the oil droplet in **(C)** over time. **(F)** Releasing a gas bubble on the superhydrophilic microstructure underwater. **(G)** Variation of the bubble in **(F)** over time. **(H)** Water droplet on the superhydrophobic microstructure. **(I,L)** Immersion of the superhydrophobic surface in water. **(J)** Placing an oil droplet on the superhydrophobic microstructure underwater. **(K)** Variation of the oil droplet in **(J)** over time. **(M)** Releasing a gas bubble onto the superhydrophobic microstructure underwater. **(N)** Variation of the bubble in **(M)** over time. Reproduced from Yong et al. (2017b) with the permission of Chen et al.

Different superwettabilities (e.g., superhydrophilicity, underwater superoleophilicity, underwater superaerophilicity, superhydrophobicity, underwater superoleophobicity, and underwater superaerophobicity) have been achieved by combing hierarchical microstructure and proper chemistry. The relationship between these different superwettabilities is summarized in Figure 2 (Yong et al., 2017b). The rough surface microstructure can amplify the natural wettability of a substrate (Yong et al., 2013a; Bellanger et al., 2014; Jiang et al., 2015; Wen et al., 2015; Su et al., 2016; Bai et al., 2020). The intrinsic hydrophilicity of a substrate can be enhanced to extreme state (i.e., superhydrophilicity) by surface microstructure; that is, the synergistic effect of the rough microstructure and high-surface-energy chemical composition produces a superhydrophilic surface (Figure 2A). Water droplets can completely wet the superhydrophilic surface microstructure at the Wenzel state in the air (Wang and Jiang, 2007; Yong et al., 2017c, 2018a). After the immersion in water, the superhydrophilicity allows the surface to be fully wet by water and the space of the microstructure to be filled with water (Figures 2B,E). The water likes being trapped by the surface microstructures, forming a tapped water cushion. As an oil droplet or a bubble is released on the superhydrophilic surfaces in water, the trapped water cushion filled in the interspaces of the surface microstructures will prevent the oil droplet/bubble from effectively touching the surface microstructure, because of the inherent repellence between water and oil droplet/bubble. The oil and bubble are only allowed to touch the peak part of the surface microstructure (Figures 2C,F). The underwater oil droplet and bubble just maintain near-spherical shapes to reach minimum free energy. Their shapes are not changed over time (Figures 2D,G). In such a three-phase (solid/water/oil or solid/water/gas) system, the underwater oil droplet (bubble) is at the underwater version of Cassie state on the structured surface (Wang and Jiang, 2007; Yong et al., 2017c, 2018a). As a result, the superhydrophilic microstructure presents superoleophobicity and superaerophobicity underwater.

By lowering surface energy (such as fluoroalkylsilane modification), the superhydrophilic microstructure can be switched to a superhydrophobic state. The superhydrophobicity results from the synergistic action of the rough hierarchical microstructures and the low-surface-energy chemical composition. Water droplets cannot wet the superhydrophobic surface and can just touch the peaks of the surface microstructure (Figure 2H). The wetting between the water droplet and the superhydrophobic surfaces belongs to the Cassie state (Wang and Jiang, 2007; Yong et al., 2017c, 2018a). An air cushion trapped in the superhydrophobic microstructure forms underneath the water droplet. The trapped air layer will develop to a surrounding air layer on the sample surface once the superhydrophobic surface is dipped into water, no matter the superhydrophobic surface faces down or up (Figures 2I,L). In a water medium, if an oil droplet is dispensed onto the superhydrophobic surfaces, the capillary action and pressure will drive the oil to enter into the trapped air layer and immediately spread out along this air gap (Figure 2J). As the oil droplet fully spread out, a very small OCA value is obtained (Figure 2K). Therefore, the superhydrophobic surface reveals underwater superoleophilicity. The behavior of an underwater bubble is very similar to that of oil droplets. Once a small bubble touches the superhydrophobic surface underwater, the gas in the bubble will enter into the trapped air layer under pressure (Figure 2M) and finally merge with the air previously trapped on the surface microstructure (Figure 2N). The bubble likes being completely absorbed by the superhydrophobic surface, resulting in the underwater superaerophilicity of the sample surface.

It is demonstrated that the superhydrophobic surface usually exhibits both underwater superoleophilicity and superaerophilicity. By contrast, the superhydrophilic surface usually shows both underwater superoleophobicity and superaerophobicity. The superhydrophilicity and superhydrophobicity of a structured surface can be transformed from one state to another state by simple hydrophobic or hydrophilic modification. As a result, the reversible transformation between different wettabilities can be achieved. For example, the laser-structured PDMS surface shows superhydrophobicity, underwater superoleophilicity, and underwater superaerophilicity (Yong et al., 2017b, 2018c). Hydrophilic modification (e.g., oxygen plasma irradiation) changes the surface wettability to superhydrophilicity, underwater superoleophobicity, and underwater superaerophobicity. Interestingly, after hydrophobic modification (e.g., storage in the air), the original superhydrophobicity, underwater superoleophilicity, and underwater superaerophilicity can completely recover. Therefore, the same rough microstructure can have various superwettabilities. The superhydrophilicity, superoleophilicity, and superaerophilicity enable the materials to have the capacity of capturing, absorbing, and collecting water droplets, oil droplets, and gas bubbles. On the contrary, the superhydrophobicity, superoleophobicity, and superaerophobicity allow the materials to greatly repel water, oil, and bubble.

## Conclusions

In conclusion, we discuss and clarify the relationship and the interconversion of superhydrophilicity, underwater superoleophilicity, underwater superaerophilicity, superhydrophobicity, underwater superoleophobicity, and underwater superaerophobicity. These different superwettabilities can be designed on a same structured surface by the combination of hierarchical surface microstructures and proper chemistry. It is revealed that the superhydrophobic surfaces usually exhibit both underwater superoleophilicity and superaerophilicity, whereas the superhydrophilic surfaces usually show underwater superoleophobicity and superaerophobicity. The superhydrophilicity and superhydrophobicity of a structured surface can be transformed from one state to another state by simple hydrophobic or hydrophilic modification. Therefore, various superwettabilities can be achieved on a same rough microstructure and reversibly convert from one state to other states. We believe that the relationship between different superwettabilities has great guiding significance for the design of superwetting materials and the applications of the artificial superwetting materials.

## Author Contributions

FC directed and supervised the research project. JY wrote the manuscript. QY and XH contributed toward significant discussions and revised the paper. All authors contributed to the article and approved the submitted version.

## Conflict of Interest

The authors declare that the research was conducted in the absence of any commercial or financial relationships that could be construed as a potential conflict of interest.
